# Supplementary material for: DNA methylation sites in early adulthood characterised by pubertal timing and development: a twin study
Source: Clin Epigenetics. 2023 Nov 10;15:181. doi: 10.1186/s13148-023-01594-7 (PMC10638786; doi:10.1186/s13148-023-01594-7)
Supplement: Supplementary file 2 — Additional file 2. Figure S1. Manhattan plots on p values of meta-analysed EWAS models on 450K and EPIC platforms on PDS at age 12 A) in males and B) females, C) combined, on PDS at age 14 in D) males and females combined, and on PA in E) males and F) females. Figure S2. QQ plots on p values of meta-analysed EWAS models on 450K and EPIC platforms on PDS at age 12 A) in males and B) females, C) combined, on PDS at age 14 in D) males and females combined, and on PA in E) males and F) females. [file 13148_2023_1594_MOESM2_ESM.pdf]

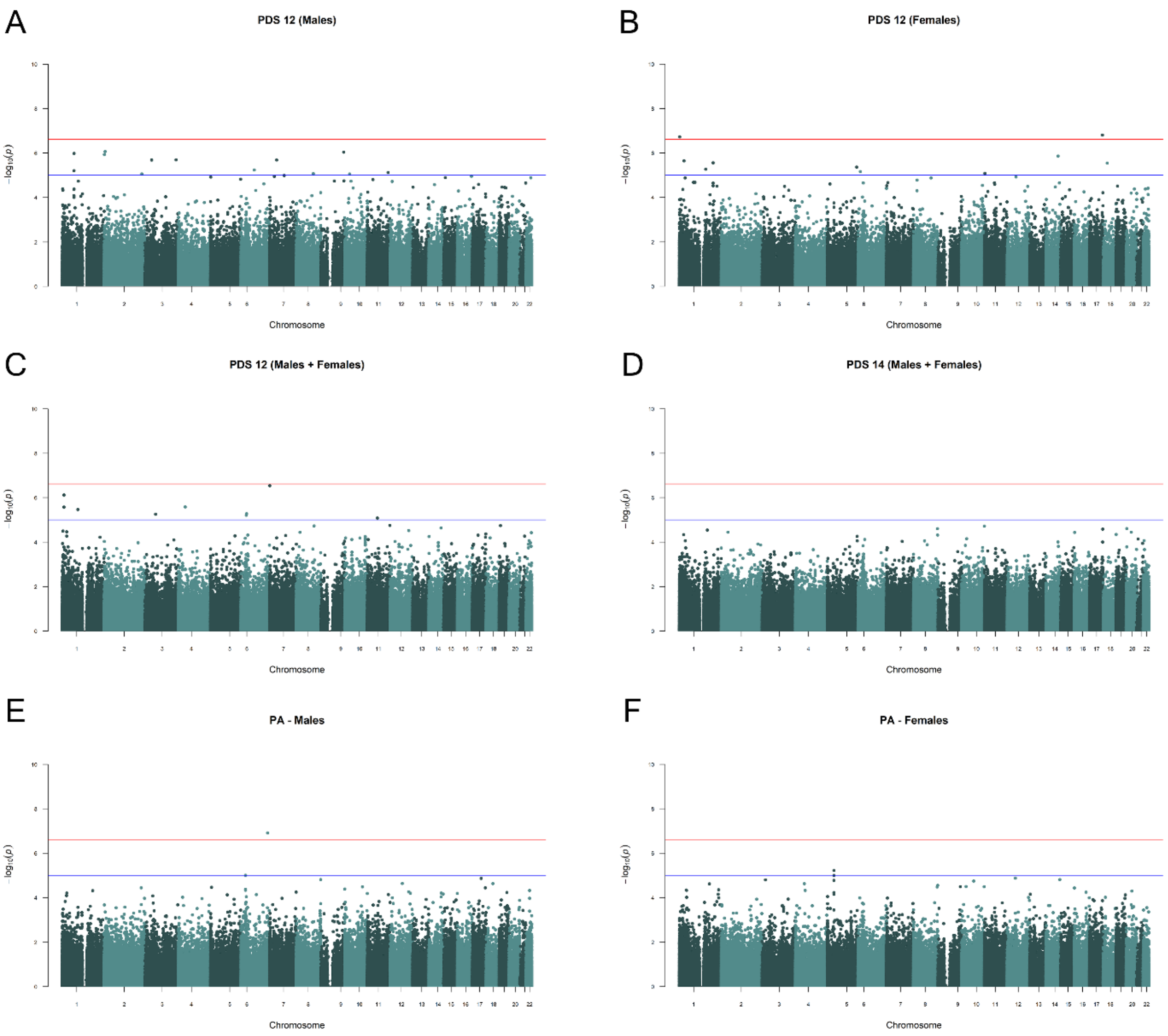

Supplementary Figure S1. Manhattan plots on p-values of meta-analysed EWAS models on 450K and EPIC platforms on PDS at age 12 A) in males and B) females, C) combined, on PDS at age 14 in D) males and females combined, and on PA in E) males and F) females

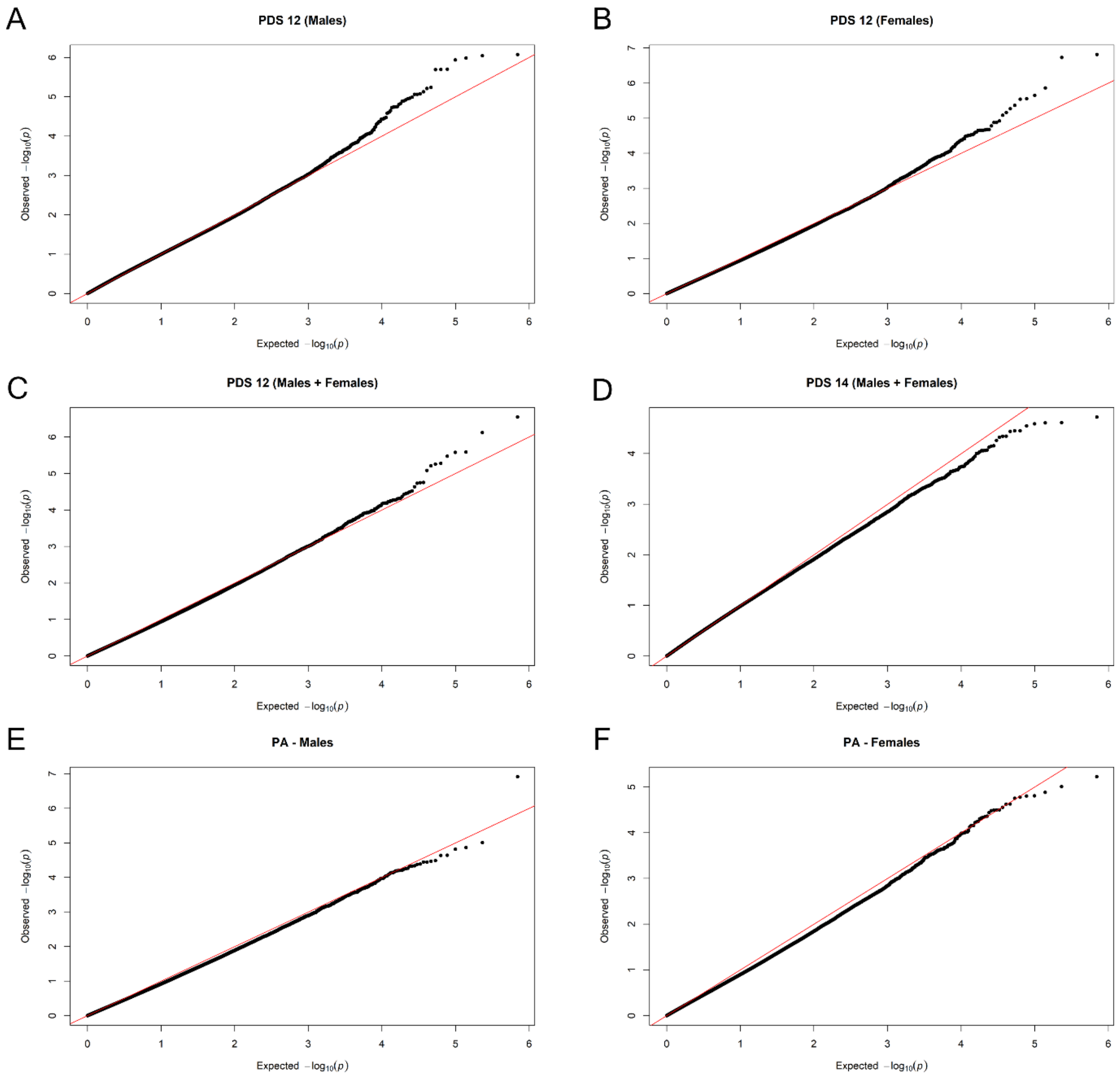

Supplementary Figure S2. QQ plots on p-values of meta-analysed EWAS models on 450K and EPIC platforms on PDS at age 12 A) in males and B) females, C) combined, on PDS at age 14 in D) males and females combined, and on PA in E) males and F) females
